# A Comparative Study of the Physicochemical Properties of a Virgin Coconut Oil Emulsion and Commercial Food Supplement Emulsions

**DOI:** 10.3390/molecules19079187

**Published:** 2014-07-01

**Authors:** Yih Phing Khor, Soo Peng Koh, Kamariah Long, Shariah Long, Sharifah Zarah Syed Ahmad, Chin Ping Tan

**Affiliations:** 1 Department of Food Technology, Faculty of Food Science and Technology, Universiti Putra Malaysia, 43400 UPM Serdang, Selangor, Malaysia; E-Mails: sweet_appie@hotmail.com (Y.P.K.); tancp@upm.edu.my (C.P.T.); 2 Biotechnology Research Center, Malaysian Agricultural Research & Development Institute (MARDI) Headquarters, Serdang, P.O Box 12301, 50774 Selangor, Malaysia; E-Mail: amai@mardi.gov.my; 3 Technical Services Center, Malaysian Agricultural Research & Development Institute (MARDI) Headquarters, Serdang, P.O Box 12301, 50774 Selangor, Malaysia; E-Mail: scha@mardi.gov.my; 4 Public Service Department of Malaysia, Block C1 & C2, Complex C, Federal Government Administrative Centre, 62510 Putrajaya, Malaysia; E-Mail: zarah@jpa.gov.my

**Keywords:** virgin coconut oil, emulsion, physicochemical properties, rheological properties, arabic gum, xanthan gum

## Abstract

Food manufacturers are interested in developing emulsion-based products into nutritional foods by using beneficial oils, such as fish oil and virgin coconut oil (VCO). In this study, the physicochemical properties of a VCO oil-in-water emulsion was investigated and compared to other commercial oil-in-water emulsion products (C1, C2, C3, and C4). C3 exhibited the smallest droplet size of 3.25 µm. The pH for the emulsion samples ranged from 2.52 to 4.38 and thus were categorised as acidic. In a texture analysis, C2 was described as the most firm, very adhesive and cohesive, as well as having high compressibility properties. From a rheological viewpoint, all the emulsion samples exhibited non-Newtonian behaviour, which manifested as a shear-thinning property. The G'G'' crossover illustrated by the VCO emulsion in the amplitude sweep graph but not the other commercial samples illustrated that the VCO emulsion had a better mouthfeel. In this context, the VCO emulsion yielded the highest zeta potential (64.86 mV), which was attributed to its strong repulsive forces, leading to a good dispersion system. C2 comprised the highest percentage of fat among all emulsion samples, followed by the VCO emulsion, with 18.44% and 6.59%, respectively.

## 1. Introduction

An emulsion is a thermodynamically unstable system, and it forms the basis of many food products [1]. The emulsion's stability is very important for ensuring its quality. This property is a chronic problem for many industries, and the stability factor is sometimes affected by the physicochemical characteristics of the gums added during the aqueous phase. A common defect in emulsions is ringing, which is mostly caused by gravitational separation, flocculation, and coalescence. The emulsion stability is highly influenced by the specific gravity, droplet size and distribution, and the rheological characteristics. The addition of hydrocolloids to the aqueous phase can yield specific rheological properties to achieve emulsion stability. Some hydrocolloids act as surface active gums (e.g., gum arabic), having the ability to form a film around the oil droplets. As a result of steric stabilisation, hydrocolloids aid in delaying this coalescence and prevent emulsion breakdown [2]. Moreover, some hydrocolloids are known to stabilise the emulsions by enhancing the viscosity of the aqueous phase (e.g., xanthan gum).

As emulsions are metastable systems, once the product leaves the factory, many environmental factors such thermal, mechanical stresses, biological action or even physical factors such as physical coarsening may occur and eventually alter the physicochemical characteristics of the product [3]. Therefore, it is also important to ensure the quality of the ingredients used, the efficiency of the homogenization, and the exclusion of microorganisms during the production of the emulsions to prevent any changes in the droplet-size distribution after prolonged storage [4]. Few aspects are important to determine the stability of the emulsion, such as the amount of emulsifiers used to produce the emulsion, oil-to-emulsifier ratio, the mobility of oil droplets, and also the interactions among the emulsion components at the interface. Furthermore, few factors such as pH, temperature, and processing conditions are found to be directly influencing the stability of the emulsion [5]. Unlike copra-derived coconut oil, virgin coconut oil possesses many health benefits and is an emerging functional food that has attracted substantial interest [6]. VCO naturally contains a mixture of medium chain fatty acids (MCFA) and long chain fatty acids (LCFA) at a ratio of 3:1 [7]. Studies have also reported that virgin coconut oil could aid in reducing total blood cholesterol, triglycerides, and phospholipids in serum and tissues [8]. Furthermore, VCO elevates the body's metabolism and energy expenditure, and it is directly converted to energy in the liver but is not stored in adipose tissue; thus, these properties suggest the potential use of VCO in anti-obesity treatments [7,9]. According to a study [10], VCO has a higher phenolic content and antioxidant activity compared to copra-derived coconut oil. Based on the benefits of this value-added functional oil, the development of a VCO-based emulsion product as a novel nutritional food supplement will indirectly increase the consumption of VCO because most consumers dislike the oily taste of pure VCO. The transformation of VCO into a more palatable and stable VCO-based emulsion product will also be an advantage for the VCO-producing industry.

The objective of this work was to evaluate the physicochemical properties of a virgin coconut oil emulsion and to compare it with other commercial food supplement emulsions. Various tests were performed on the emulsions, namely, a droplet size distribution profile, pH analysis, texture analysis, rheological analysis, microscopic observation, zeta potential analysis, and proximate analysis to gauge their quality.

## 2. Results and Discussion

### 2.1. Proximate Analysis

**Figure 1:**
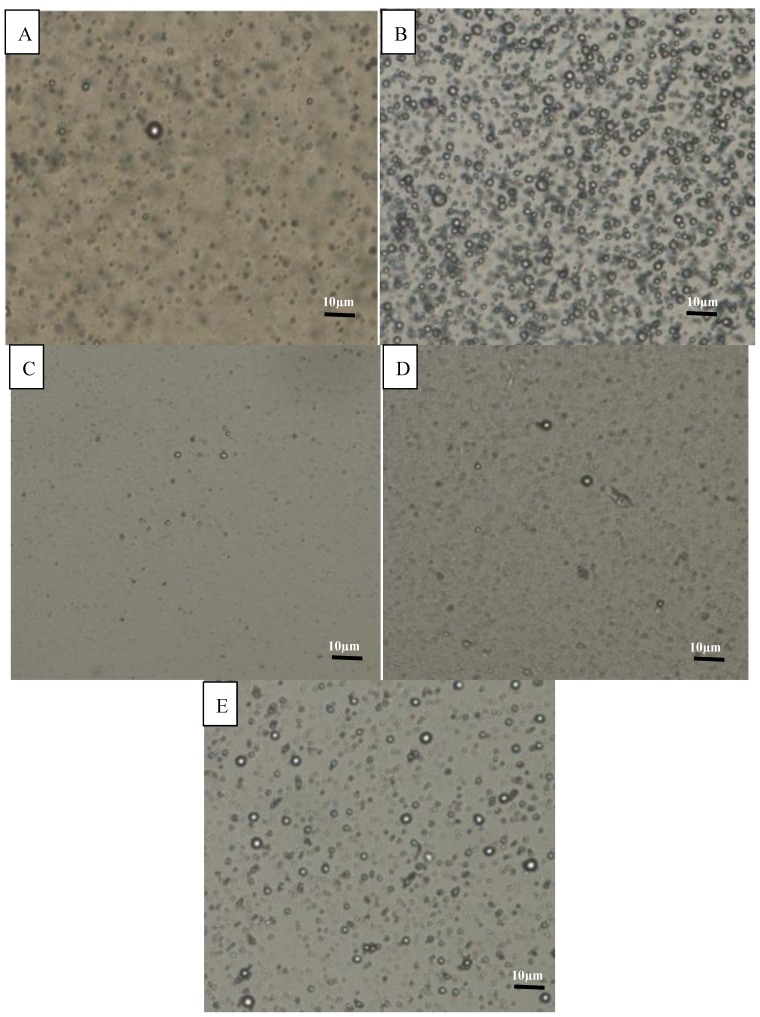
Microscopic observation of commercial emulsion samples (**A**) C1, (**B**) C2, (**C**) C3, (**D**) C4, and (**E**) virgin coconut oil emulsion. Scale bar in all images represents 10 µm.

Table 1 shows proximate analysis results for all the food emulsion samples. C2 was shown to contain the most fat of all the food emulsion samples. This finding could be justified by the strong oil odour in C2. Additionally, Figure 1 also illustrates that C2 had the highest number of fat globules among the emulsion samples.

**Table 1:** Proximate analysis of the commercial emulsion samples and virgin coconut oil emulsion.

Sample	% Fat	% Protein	%Moisture	% Ash	% Crude fibre	% Carbohydrate
C1	0.26 ± 0.04 ^a^	0.08 ± 0.02 ^b^	54.59 ± 0.36 ^b^	2.76 ± 0.07 ^c^	0.00 ± 0.00 ^a^	42.33 ± 0.25 ^d^
C2	18.44 ± 0.35 ^c^	0.00 ± 0.00 ^a^	65.97 ± 0.27 ^c^	1.63 ± 0.10 ^b^	0.00 ± 0.00 ^a^	13.96 ± 0.55 ^b^
C3	0.11 ± 0.03 ^a^	0.07 ± 0.00 ^b^	68.03 ± 0.21 ^d^	3.43 ± 0.05 ^d^	0.00 ± 0.00 ^a^	28.37 ± 0.17 ^c^
C4	0.45 ± 0.01 ^a^	0.07 ± 0.02 ^b^	31.14 ± 0.23 ^a^	0.09 ± 0.00 ^a^	0.00 ± 0.00 ^a^	68.24 ± 0.22 ^e^
VCO	6.59 ± 0.01 ^b^	0.00 ± 0.00 ^a^	89.55 ± 0.23 ^e^	0.11 ± 0.02 ^a^	0.00 ± 0.00 ^a^	3.75 ± 0.14 ^a^

Data were expressed as mean ± standard deviation (n = 6). Mean values with different superscripts in the same column are significantly different at *p* < 0.05.

All emulsion samples had very little protein content. Because of the absence of a specific nitrogen: protein conversion factor for emulsion, the protein was estimated by multiplying the values obtained for the percentage nitrogen by a default value of 6.25. Moreover, all of the emulsion samples possessed a high moisture level, with values ranging from 31.14% to 68.03%. This phenomenon may cause the samples to be more susceptible to microbial spoilage; therefore, combating this issue should be taken into consideration. No emulsion samples contained crude fibre, and they all yielded a negligible amount of ash. The main carbohydrate source in the emulsion samples may have originated from the hydrocolloid source or added sweetener.

### 2.2. Droplet Size Distribution

Droplet size measurements delivered information about the emulsion stability [11]. Most food emulsion properties (e.g., physical and microbiological stability, rheology and optical characteristics) largely depended on the emulsion microstructure; for instance, the droplet size distribution [12]. The production and control of emulsions with a narrow size distribution has attracted considerable attention from the food, pharmaceutical, and cosmetic industries in recent years. The average droplet sizes (d_4,3_) of the studied emulsions are shown in Table 2. The droplet movement of the large particle in C1 might induce flocculation [13]. A small droplet size was favoured in emulsions because the decrease in droplet size would reduce the number of lipid molecules per droplet and, thereby, increase the amount of surface-active compounds adsorbed at the interface, exhibiting greater physical stability. Moreover, the smaller droplet size increased the number of particles and led to an increase in particle-particle interactions, which also led to an increase in emulsion viscosity.

**Table 2:** pH, droplet size and zeta potential of the commercial emulsion samples and virgin coconut oil emulsion.

Sample	pH	Droplet size (µm)	Zeta potential (mV)
C1	2.52 ± 0.02 ^a^	217.00 ± 14.96 ^b^	−27.07 ± 0.68 ^d^
C2	2.85 ± 0.04 ^c^	15.48 ± 0.43 ^a^	−34.53 ± 0.25 ^c^
C3	2.63 ± 0.01 ^b^	3.25 ± 2.25 ^a^	−23.17 ± 0.65 ^d^
C4	3.45 ± 0.02 ^d^	19.54 ± 2.51 ^a^	−52.77 ± 3.35 ^b^
VCO	4.38 ± 0.01 ^e^	9.57 ± 3.06 ^a^	−64.86 ± 0.25 ^a^

Data were expressed as mean ± standard deviation (n = 6). Mean values with different superscripts in the same column are significantly different at *p* < 0.05.

**Figure 2:**
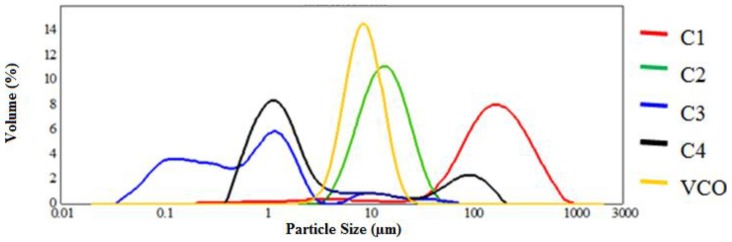
Droplet size distribution of commercial emulsion samples (C1, C2, C3, C4) and virgin coconut oil emulsion.

In this context, C3 and VCO emulsions possessed small droplet sizes. However, based on the droplet size distribution shown in Figure 2, only C2 and VCO emulsions showed sufficiently small droplet size and were monomodal with no evidence of flocculation. Initiation and propagation would be limited as surface-active compounds acted as a barrier to the penetration and diffusion of pro-oxidants or interfered with oxidation [14]. The droplet size of the emulsion directly influenced the quality of the end product. The system chosen to produce the VCO emulsion was the rotor-stator system. This system was widely used for the emulsification of liquids with medium to high viscosities. High fluid acceleration led to the mechanical impingement of emulsion against the wall, creating a force that reduced the droplet size of the emulsion. In addition, the rapid rotation of the rotor generated shear stress in the gap between the rotor and stator that reduced the droplet size distribution [11].

### 2.3. pH Measurement

The pH of the emulsion samples fell in the acidic range, varying from 2.52 to 4.38. These acidic emulsion samples were thought to be effective in inhibiting microbial activity and eventually improving a product's shelf stability [15].

### 2.4. Zeta Potential

As shown in Table 2, the zeta potential values of the emulsion samples varied from −23.17 mV to −64.86 mV. However, for C1 with the mean droplet size of 217 µm, the zeta potential of −27.07 mV may not be accurate as the maximal size range of the apparatus for measuring correctly the zeta potential is 100 µm. With the addition of gum arabic and xanthan gum, VCO emulsion was negatively charged at pH 4.4. The VCO emulsion exerted the largest negatively charged zeta potential, which also meant the strongest repulsive forces were found between particles in the emulsion relative to the commercial samples. A prior study reported that the zeta potential of the emulsion droplets was strongly influenced by the final pH, type of polysaccharide used, and the polysaccharide concentration [16].

### 2.5. Texture Profile

These measurable quantities were relevant to the product's mouthfeel because they have been shown to relate directly to the experience of the consumer at the time of chewing and swallowing food. The four main parameters for texture analysis were firmness, cohesiveness, adhesiveness, and compressibility. The resulting data indicated a notable influence of the oil volume fraction towards textural parameters [17]. The hardness attribute described a product that displayed substantial resistance to deformation or breaking. This perceived force was required for the molars to break the sample during the first bite. The percentage of oil phase was likely to impact the emulsion hardness because increasing the oil phase percentage increased the emulsion hardness [17]. 

In addition, adhesiveness was important to gauge the force required to remove the food that had bonded to hands during handling or to the roof of the mouth during eating. Furthermore, the cohesiveness indicated the extent of the deformation and destruction when a load was applied to the product. The compressibility was also vital for determining the sample’s ability to withstand pressure. The lower the firmness of the emulsion, the easier it would be for our mouth to break the sample and swallow. On the other hand, the higher the compressibility, the better the emulsion sample’s ability to withstand pressure exerted during processing. In addition, the lower the cohesiveness, the better the emulsion because the particles tended to disperse properly in the continuous phase. This property could also reduce the risk of flocculation and coagulation.

Last but not least, the lower the adhesiveness, the better the emulsion because the emulsion did not adhere strongly to the packaging material, which eased the pouring of the emulsion out from the packaging. Cohesion and adhesion forces were regarded as important driving forces behind the dispersion instability mechanisms. It was worth pointing out that the VCO sample texture attributes fell within the range of the commercial samples (Table 3). VCO emulsion could be described as less firm, less cohesive and adhesive and having high compressibility.

**Table 3:** Texture properties of the commercial emulsion samples and virgin coconut oil emulsion.

Sample	Firmness (g)	Compressibility (g s^−1^)	Cohesiveness (g)	Adhesiveness (g s^−1^)
C1	17.12 ± 0.18 ^b^	231.70 ± 33.15 ^a^	−5.50 ± 0.16 ^b^	−3.26 ± 0.79 ^bc^
C2	27.50 ± 0.24 ^c^	387.99 ± 33.03 ^c^	−17.15 ± 0.68 ^a^	−14.51 ± 2.04 ^a^
C3	13.93 ± 0.02 ^a^	257.36 ± 6.26 ^ab^	−2.95 ± 0.18 ^d^	−0.86 ±0.06 ^c^
C4	17.13 ± 0.03 ^b^	280.69 ± 13.71 ^ab^	−5.64 ± 0.21 ^b^	−3.02 ± 0.15 ^bc^
VCO	16.97 ± 0.13 ^b^	304.39 ± 12.81 ^b^	−4.52 ± 0.20 ^c^	−3.58 ± 0.07 ^b^

Data were expressed as mean ± standard deviation (n = 6). Mean values with different superscripts in the same column are significantly different at *p* < 0.05.

### 2.6. Rheological Profile

**Figure 3:**
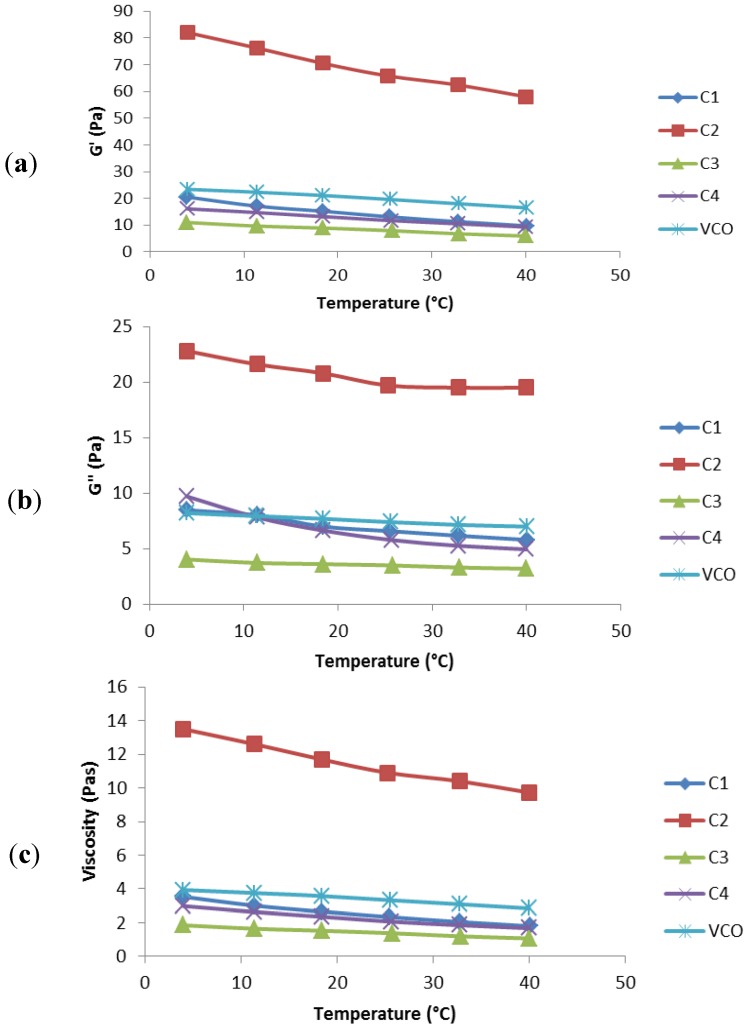
(**a**) Storage modulus G' (Pa) *vs.* temperature sweep (°C), (**b**) loss modulus G'' (Pa) *vs.* temperature sweep (°C), and (**c**) viscosity η (Pa.s) *vs.* temperature sweep (°C) data for commercial emulsion samples (C1, C2, C3, C4) and virgin coconut oil emulsion.

Stress rheometry was commonly used as a method to assess flocculation because the results were generally consistent with the creaming behaviour of emulsions [18]. In addition, the rheological characteristics were able to reveal the macroscopic behaviour of the emulsion system with respect to the microstructural organisation [19]. From the temperature sweep test, the viscosities of the emulsion samples were shown to decrease in the following order C2 < VCO emulsion < C1 < C4 < C3 (Figure 3). Therefore, the viscosity value of the virgin coconut oil emulsion fell in a range from the lowest viscosity commercial sample to the highest viscosity commercial sample. C2 was determined to have the highest viscosity, G' (energy storage) and G'' (loss of energy), which required higher forces to initiate and continue flow. The high viscosity in C2 might be due to the high oil volume fraction which present in the emulsion as shown in Table 1.

Besides that, the rheology of the emulsion was sometimes dominated by the rheology of its continuous phase, in which high viscosity could greatly delay the creaming of the droplets [20]. Noticeably, there was no significant change in the emulsion viscosities over the temperature changes except for C2. This finding may indicate that the emulsions did not display flocculation. It could also be deduced that the emulsion viscosity and the strength of the emulsion system increased as the oil phase percentage increased, but decreased slightly with temperature [17,19]. No significant changes (*p* > 0.05) were detected in G' (elastic component) and G'' (viscous component) upon increase of temperature in C1, C3, C4, and VCO-based emulsion samples. This could indicate good emulsion stability of the emulsion systems. However, a gradual decrease in G' and G'' was observed in C2 and this might be due to the higher viscosity of C2 emulsion compared to the other emulsion samples. Moreover, G' remained higher than G'' in all emulsions throughout the temperature sweep test. This phenomenon indicated the elastic properties in all emulsions.

**Figure 4:**
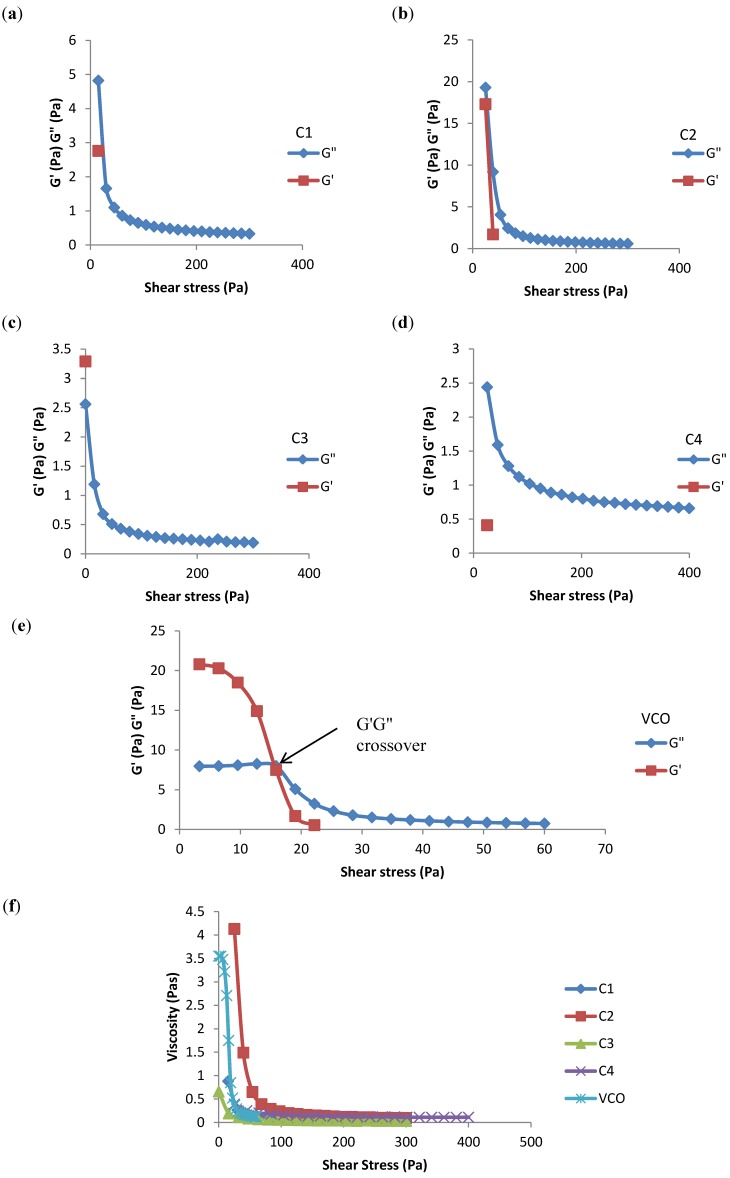
Storage modulus G' (Pa) and loss modulus G'' (Pa) *vs.* shear stress (Pa) data for (**a**) C1; (**b**) C2; (**c**) C3; (**d**) C4; (**e**) virgin coconut oil emulsion and (**f**) viscosity η (Pa.s) *vs.* shear stress (Pa) for commercial emulsion samples and virgin coconut oil emulsion.

As indicated by the amplitude sweep plot (Figure 4), there was a decreased viscosity among all emulsions with increasing shear rate. This finding clearly indicated the shear-thinning (pseudoplastic) behaviour of non-Newtonian emulsion samples [1,2,13,21]. In other words, the viscosity magnitude obtained in these regions was always shear rate-dependent [22]. However, no G' (storage modulus) and G'' (loss modulus) crossovers were observed in all the commercial emulsion samples relative to the crossover exhibited by the VCO emulsion sample. This finding suggested that the mouthfeel of the VCO emulsion was better than that of the commercial sample.

**Figure 5:**
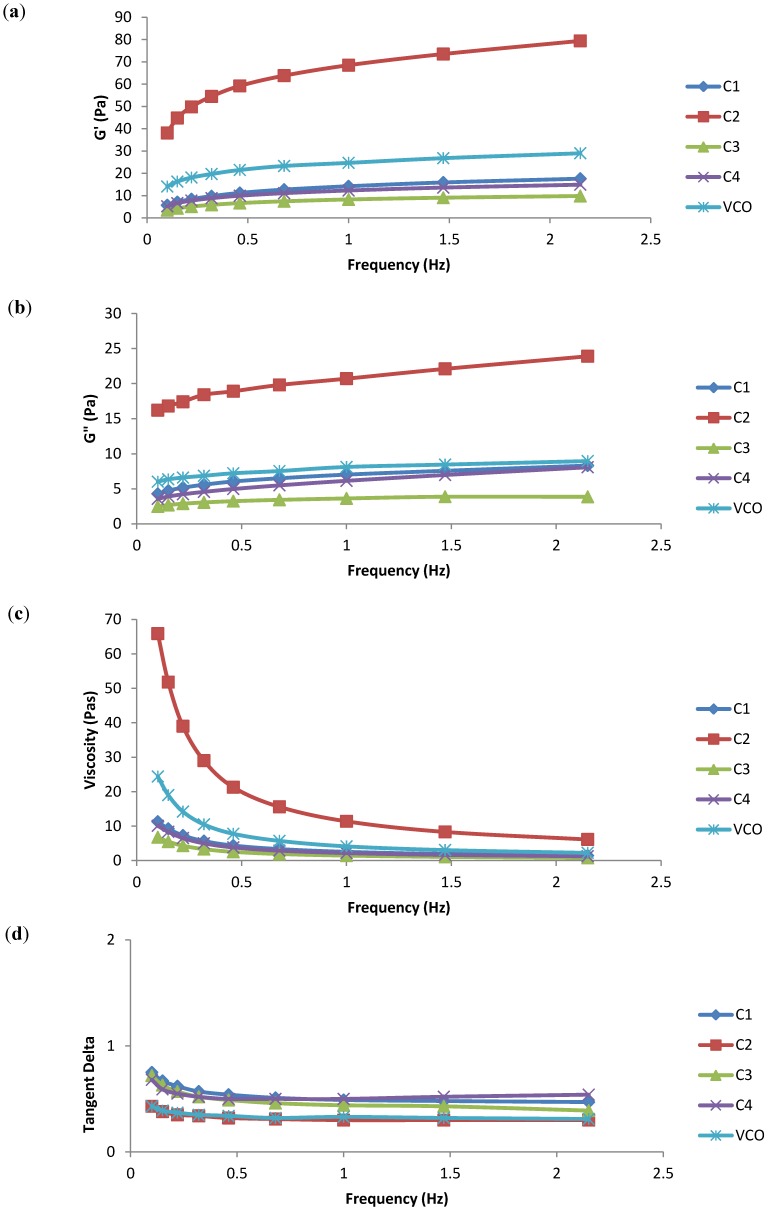
(**a**) Storage modulus G' (Pa) *vs.* frequency sweep (Hz), (**b**) loss modulus G'' (Pa) *vs.* frequency sweep (Hz), (**c**) viscosity η (Pa.s) *vs.* frequency sweep (Hz), and (**d**) loss tangent (tan δ) *vs.* frequency sweep (Hz) data for commercial emulsion samples (C1, C2, C3, C4) and virgin coconut oil emulsion.

All emulsions showed an elastic solution behaviour with a lower G'' than G' throughout the frequency sweep plot (Figure 5). In spite of this finding, the report revealed that when the elastic modulus (G') was larger than the viscous modulus (G''), the emulsions with a predominant elastic behaviour would demonstrate greater stability [2,22].

Wiacek *et al.* [23] also suggested that emulsions with good elasticity would provide an excellent barrier to collision coalescence. Based on the frequency sweep test, the G'' of all the samples were shown to increase in parallel with G' but at a relatively lower magnitude than G'. This finding implied the existence of a gel or network structure in all the emulsion samples [24,25]. However, these emulsions were weakly structured systems, as a consequence of the development of a three-dimensional network with rheological “units” linked by weak bonds [19]. The emulsions correspondingly behaved as solids under small deformations, while they flowed under large stresses owing to the breakage of weak bonds.

The emulsions exhibited a power law relaxation mechanism from a rheological point of view [22]. On the other hand, the viscosity decreased with an increasing frequency sweep. These shear-thinning emulsions had a high viscosity at low shear rates but decreased dramatically as the shear increased. This property was vital to ensure an easy emulsion flow when poured from a container because the droplets were prevented from creaming [2]. Tangent δ acted as an indicator of whether the samples behaved as viscous (liquid) or elastic (solid). In this frequency sweep study, it was worth noting that all tangent δ values of the emulsion samples fell below 1, subsequently confirming the elastic behaviour of these emulsions [24].

## 3. Experimental Section

### 3.1. Materials

Commercial food supplement emulsion samples were purchased from a local pharmacy in Selangor, Malaysia, and were designated C1, C2, C3, and C4. Virgin coconut oil was purchased from Cocorosco Sdn. Bhd. (Johor, Malaysia). All the solvents and chemicals used for this analysis were of analytical grade. Potassium sorbate, citric acid, and xanthan gum were purchased from the Meilun Food Chemical Sdn. Bhd. (Selangor, Malaysia). Stevia and gum arabic were purchased from the SteviaSugar Corporation (M) Sdn. Bhd. (Kuala Lumpur, Malaysia) and the Markaids (M) Sdn. Bhd. (Selangor, Malaysia), respectively. Modified starch was obtained from San Soon Seng Food Industries Sdn. Bhd. (Selangor, Malaysia). Flavouring agent was obtained from the Reka Nutrition Sdn. Bhd. (Selangor, Malaysia).

### 3.2. Physicochemical Properties Analysis

A virgin coconut oil emulsion and commercial food supplement emulsion samples were subjected to various physicochemical tests, namelyproximate analysis, microscopic observation, droplet size analysis, pH analysis, zeta potential, texture analysis, and rheological analysis. All the results were reported as the means of triplicate measurements (mean ± standard deviation).

#### 3.2.1. Preparation of Virgin Coconut Oil Emulsion

A VCO emulsion formulation of 200 mL was prepared according to the following proportions of VCO/gum arabic/xanthan gum/modified starch: (9.4:1.3:1:1.3). To prepare the VCO emulsion, the polysaccharide dispersion was first prepared. Stevia (1%, w/v), potassium sorbate (0.1%, w/v), citric acid (0.08%, w/v) and modified starch were dispersed in deionised water at 40 °C. Potassium sorbate and citric acid were selected as the agents to protect against microbial growth. A sufficient amount of a natural sweetener, stevia, was introduced into the VCO emulsion formulations to mask the taste of the VCO in a healthy way, without contributing to the bitterness of the after taste. Gum arabic was slowly added to the aqueous phase and mixed continuously using a magnetic stirrer until it was fully dissolved. Then, xanthan gum was added. The mixture was further stirred to ensure complete dissolution. The VCO and flavouring (0.5%, w/v) were added dropwise to the continuous phase. The droplet size of the resulting crude emulsion was further reduced to fine droplet size by using a high-shear homogeniser with a round hole shear screen as a dispersing tool. The crude emulsion was homogenised at 7,000 rpm for 1.5 min. The resulting fine emulsion was allowed to cool before analysis.

#### 3.2.2. Proximate Analysis

Fat content was determined by the modified Mojonnier method with an ether extraction (AOAC 989.05). Protein content was determined by the Kjeldahl distillation method (AOAC 981.10). A correction factor of 6.25 was applied to convert the % nitrogen to % protein. The sample was ashedin a muffle furnace at 800 °C to determine the ash content (AOAC 930.05). The crude fibre content of the emulsion was determined based on the acid-alkaline neutralisation method. The moisture content of the emulsion samples was determined with a moisture analyser (MX-50, A&D, Tokyo, Japan).

#### 3.2.3. Microscopic Observation

A light microscope (Eclipse 80i Binocular, Nikon, Melville, NY, USA) equipped with a CCD camera (Nikon 5 megapixel, Kanagawa, Japan) connected to digital image processing software (NIS-Elements Basic Research, Nikon Instruments, Melville, NY, USA) was used to observe the internal structures of the emulsion samples. A thin emulsion layer was spread on the microscope slide and covered with a cover clip before viewing it under the microscope. The samples were analysed using a magnification of 400× at room temperature.

#### 3.2.4. Droplet Size Distribution

The emulsion droplet size distribution was measured by using a Malvern Mastersizer MS 2000 static laser light-scattering analyser (Malvern, Worcestershire, UK). This instrument measured the angular dependence of the light intensity when scattered from a dilute emulsion. A refractive index ratio of 1.46 was adopted. Samples were then diluted in a sample cell containing water until 15 to 20% of the incident light was absorbed. Measurements were performed in triplicate and the results were reported as the means. The emulsion droplet sizes were expressed as the volume mean diameter:

D[4,3] (µm) = ∑*d^4^/∑d^3^* (1)

where *d* is the diameter (µm) of a droplet*.*

#### 3.2.5. pH Measurement

A 7.5% (w/v) virgin coconut oil emulsion was prepared and cooled to room temperature (25 ± 0.5 °C). A digital pH meter (Mettler Toledo, Columbus, OH, USA) was used to measure the pH values of the food emulsion samples.

#### 3.2.6. Zeta Potential

The emulsion samples were diluted (1:100) to measure their conductivities with a Malvern Zetasizer (Malvern series ZEN 3500). This test was carried out by measuring the electrophoretic mobility of dispersed particles in a charged field. When the zeta potential was relatively high (30 mV or more), the repulsive forces exceeded the attractive London forces. The particles were dispersed and the system was deflocculated. To avoid multiple scattering effects, the emulsion sample was diluted with deionised water (0.5 mL/50 mL) prior to analysis and was then directly placed into the module. The measurement range of a Malvern Zetasizer was expressed in mS/cm to indicate conductivity. The conductivity measurements were carried out immediately after sample preparation. The measurements were carried out in fully automatic mode and were reported as the average of three separate injections, with three readings per injection. The averages of the triplicate values were used as the response values for the conductivity.

#### 3.2.7. Texture Profile

Emulsion texture measurements were carried out with the TA.XT2i texture analyser (Stable Micro Systems, New York, NY, USA) using a 5 kg load cell. A 1” stainless spherical probe was used. One cycle was applied at a constant crosshead velocity of 2 mm/s. Once a 5 g trigger was detected at the surface of the sample, the probe penetrated the emulsion at 1 mm/s until reaching 75% of the total strain. After the probe had reached this distance, it withdrew at 10 mm/s to its start position above the emulsion surface. Various texture attributes were determined from the resulting force-time deformation curve, *i.e.*, firmness, cohesiveness, adhesiveness, and compressibility.

#### 3.2.8. Rheological Profile

The viscoelastic properties of the food emulsion samples were analysed using a dynamic controlled stress rheometer (RS600, Thermo Electron Corporation, Osterode am Harz, Germany). Parallel-plate geometry was used at a 35 mm diameter and a gap of 1 mm. The measuring device was equipped with a temperature unit in which a programmable water bath (K20, Haake, Waltham, MA, USA) with a Universal Temperature Controller (Haake) was used to ensure precise and stable temperature control during measurements. Frequency sweep tests were carried out at frequencies ranging from 0.1 to 2.15 Hz, with the temperature maintained at 25 °C and a constant strain of 1%. The storage modulus (G'), loss modulus (G''), viscosity (η), and loss tangent (tan δ) *versus* the frequency were measured for all samples. A dynamic stress sweep was carried out on all samples at a range varying from 0.1 to 300 Pa, and the frequency was maintained at 1 Hz and the temperature was held at 25 °C. The storage and loss modulus crossover and the viscosity *versus* stress were analysed for all emulsion samples. A temperature sweep test was also conducted, with the temperature ranging from 4 to 40 °C, while the frequency and strain were kept constant at 1 Hz and 1 Pa, respectively. The storage modulus, loss modulus, and viscosity *versus* temperature were measured for all samples.

#### 3.2.9. Statistical Analysis

The experimental results were analysed using Minitab software (Minitab Version 14.1, Minitab Pty Ltd, Sydney, NSW, Australia). All data were expressed as the means ± standard deviations of triplicate measurements. A one-way analysis of variance (ANOVA) at the 5% significance level was used to determine significant differences (*p* < 0.05) between the means.

## 4. Conclusions

In this study, the physicochemical properties of VCO emulsions were investigated and compared to the commercial emulsion products (C1, C2, C3, and C4). The VCO emulsions and commercial emulsion samples were significantly different (*p* < 0.05) in terms of droplet size, pH, texture profile, rheological profile, zeta potential, and product chemical composition. The VCO emulsion was reported to have a small droplet size (9.573 µm) that improved the shelf stability of the product. Additionally, its pH of 4.38 fell in the acidic range and was quite effective at the inhibition of microbial activity. VCO emulsions exerted good textural properties and could be described as less firm, less cohesive and less adhesive, and having high compressibility. Moreover, they exhibited shear-thinning behaviour and relatively high G'G'' crossover, which yielded a better product mouthfeel. The large negatively charged zeta potential in the VCO emulsion also demonstrated that the product has a good dispersion system. In conclusion, based on all the reported parameters, the VCO emulsion was characterised as a stable emulsion with good textural properties and could be used as a novel food supplement to increase the consumption of VCO among consumers.
